# Tetryl exposure: forgotten hazards of antique munitions

**DOI:** 10.1186/s40557-016-0102-7

**Published:** 2016-04-08

**Authors:** Walla A. Alfaraj, Brian McMillan, Alan M. Ducatman, Charles L. Werntz

**Affiliations:** West Virginia University Institute for Occupational and Environmental Health, Morgantown, West Virginia USA; Saudi Arabia Cultural Mission, Fairfax, West Virginia USA; School of Medicine, West Virginia University, Morgantown, West Virginia USA; School of Public Health, West Virginia University, Morgantown, West Virginia USA; 222 N Charles St, Baltimore, MD 21201 USA

**Keywords:** Tetryl, Munitions, West Virginia, Occupational safety

## Abstract

**Background:**

Older yet still abundant munitions such as tetryl present easily forgotten health hazards and associated needs for worker protection.

**Case presentation:**

Symptoms and findings from 22 workers who were exposed to tetryl are summarized.

**Conclusions:**

This study highlights the health hazards from exposure to tetryl. Occupational health professionals need to maintain vigilance to protect workers from the risks of handling older munitions.

## Background

Tetryl, or 2,4,6-trinitrophenyl methyl nitramine, is an explosive similar to trinitrotoluene (TNT) and was used extensively during World War I and II as a detonator and booster in munitions [1]. Tetryl’s stability over time made it a desirable explosive for military applications. U.S. Post-WWII production was limited to one plant, and ended in 1973 [2]. Tetryl is still used internationally in land mines. Today the most likely contact with tetryl in the U.S. is workers involved in disposal, destruction, and cleanup of discarded munitions [3]. In 2007, the US Department of Defense proposed a beneficial use of this hazardous military waste, creating a novel plan to dispose of demilitarized tetryl as part of surface mining of coal. Surface mine operations usually involve drilling a row of holes that are filled with explosives (generally ammonium nitrate-fuel oil (ANFO)) to blast away the “overburden”. Handling of ANFO does not cause any know chronic disease [4] but that same safety does not apply to older munitions such as Tetryl.

In 1943 industrial hygienists determined that tetryl concentration of 1.5 mg/mg/m3 was considered to produce no health effects [4, 5]. The current OSHA permissible exposure limit [PEL] remains at 1.5 mg/m and has the [skin] notation [6, 7]. There is an “advisory” OSHA guideline for tetryl exposure, no other OSHA regulations exist [7]. Here we report the health symptoms and findings of 22 surface mineworkers exposed during a tetryl disposal operation in West Virginia, USA, and who then presented to the university clinic. The goal of presenting this case series is to emphasize the importance of recognizing previously known hazards before historic knowledge of safe handling older munitions is lost.

Following verbal encouragement from Department of Defense personnel, two surface coalmines in West Virginia participated in a novel beneficial use of tetryl. Tetryl munitions, stored at a demilitarizing facility, were loaded onto trucks and transported to the mine site. Workers employed by contract blasting operators were instructed to slowly add tetryl into the modern conventional blasting material (ANFO) as it flowed into the drilled holes. The goal was 1:10 tetryl to ANFO ratio. The loading, unloading, and pouring operations were all reported to be consistently dusty. Heavy equipment operators were tasked to remove the detonation-loosened overburden, and overburden removal was reported to be intermittently dusty, with pre-and-post detonation worker’s reporting yellow tetryl dust blowing into their breathing zones.

Before operations began, workers and explosives contractors were instructed that tetryl exposure was safe. No safety precautions were needed beyond those normally used for ANFO. Dermal and respiratory Personal Protective Equipment (PPE) were not recommended or provided to the workers. Workers did not shower on-site, and most wore their work clothing home. Nearly all of the patients in this cohort reported tracking tetryl dust into their vehicles and homes. Twelve of the workers also reported that they took empty WWII vintage ammunition boxes home as souvenirs. One asthmatic spouse reported increased asthma symptoms when washing work clothes during the period of tetryl use at the mine.

This tetryl disposal program ended after a tetryl-containing “shot” spontaneously ignited (EVENT A), generating a brownish-red cloud (presumed to be oxides of nitrogen) described to reach a height of 150 feet in February 2007. Dust from previously deployed tetryl remained on site for some time following. Before the operation ceased, the total tetryl exposure amount used in blasting (or lost as dust) was estimated to be 300,000-370,000 lbs. (136,000-168,000 kg) over the course of three months. Eventually, the military reassumed control of the remaining tetryl and removed it from the area.

## Case presentation

Our clinicians were notified by the West Virginia Poison Center (WV-PC) following EVENT A, and receipt of health inquiries from workers. Referrals to the university clinic came from the WV-PC, as well as primary care and consulting physicians in the community. Twenty-two concerned workers appeared in the university clinic between February and April 2007. All patients were seen >1 week following EVENT A. However, post incident industrial hygiene analysis made it clear that residual dust remained on site, as well as in vehicles. There may also have been residual tetryl in some homes, but no analysis was done.

An evaluation protocol based on the known effects of tetryl included a comprehensive history, physical examination, complete blood count, liver function and renal function testing, as well as pulmonary function tests (PFTs) with bronchodilators if indicated by obstructive findings. Response to bronchodilator was graded by the American Thoracic Society criteria. If a patient complained of airway symptoms and baseline spirometry was normal, then methacholine challenge testing (MCT) was performed. MCT results were graded as severe (>20 % reduction in flow at <0.3 mg), moderate (0.3–1.7 mg), mild (1.7-8.0 mg), and normal (>8 mg). After obtaining an Institutional Review Board exemption for the presentation of summary data, charts were reviewed, and summary statistics complied. The cohort represented three types of work exposure: twelve workers handled tetryl prior to normal detonations, eight workers were exposed to airborne dust after detonation; and 10 workers were present at EVENT A. The groups overlap, there were 22 total workers seen. Twelve workers wore cotton gloves; one worker requested a respirator and was issued a cloth mask. No chemical or dust protective equipment was recommended or provided.

Shown in Table 1, the most common health complaints were headache and dry cough, pertaining to most workers. Shortness of breath (14 workers) was common, followed by rash (10), sore throat (9) nose bleed or bloody rhinorrhea (9) nasal irritation (7), skin irritation (7), and fatigue (7). Six workers reported experiencing yellow skin discoloration, but skin discoloration (primarily of hands) could also be seen at the time or presentation on workers who did not report this symptom Less commonly reported symptoms were insomnia, nausea, and palpebral or orbital edema. Of these symptoms, skin discoloration and cough persisted and could be independently verified by examining clinicians. These are consistent with the literature concerning tetryl exposure.

**Table 1:** The Symptomology Reported By Workers Exposed To Tetryl (*n* = 22)

	N (%)
Skin and mucous membrane	
Rash	10 (45 %)
Skin irritation	7 (32 %)
Yellow skin discoloration	6 (27 %)
Respiratory symptoms	
Dry cough	17 (77 %)
Sore throat	9 (41 %)
SOB	14 (64 %)
Nasal irritation	10 (45 %)
All others	
Nausea	3 (14 %)
Insomnia	5 (23 %)
Fatigue	7 (32 %)
Nose bleeds	6 (27 %)
Orbital edema	2 (09 %)
Headache	17 (77 %)

Jaundice, myeloid-suppression and kidney diseases reported in the historic literature were not seen in this cohort. However, liver function tests were abnormal at the time of evaluation in three workers who did not consume alcohol; unknown if this relates to their exposure. Methacholine challenge test results allowed better characterization of pulmonary effects than prior cohorts and did support the existence of occupational asthma as the cause of persistent cough.

All workers’ compensation claims for this exposure were initially denied, presenting workers with an economic disincentive for timely follow-up.

## Discussion

The symptoms reported by these workers are consistent with the distant past literature. Yellowish coloring of exposed skin and hair was common [1, 3]. Tetryl workers were historically known as “canaries” [2, 7]. Skin and hair discoloration can start within 1–3 days of exposure and may vary in shade becoming a deeper orange upon exposure to sunlight [8, 9]. Oily skin or heavy sweat may intensify this effect [9]. Discoloration is not independently indicative of further symptoms but confirms exposure [2]. Laboratory testing did not reveal any findings not described in the past.

Shortness of breath and airway tightness including cough, nasal, and throat irritation, and sneezing were the most significantly persistent reports and we found pulmonary function tests to be unrevealing in most workers. Bronchial irritation, inflammation, and asthma-like symptoms were historically common, with symptoms generally resolving within three weeks after removal from exposure [2, 6], no evidence of parenchymal pulmonary pathology was found in studies of 4000 munitions workers, with study authors theorizing that the large size of the tetryl crystals made them unlikely to enter the smaller bronchioles. Historically, patients with severe sensitization were permanently removed from all exposure while those who returned to work following mild reactions were found to have “working tolerance” in 60 % of workers [2]. Chemical hepatitis may have occurred in our series. Although “liver function” testing may underestimate liver damage in populations, we believe that any hepatitis was mild and reversible, but we lack follow-up information. Tetryl historically has been associated with general malaise, irritability, insomnia, headaches, epigastric pain, nausea, anorexia, vomiting, jaundice, liver function abnormalities, irregular menstruation, leukocytosis, and anemia [2]. Among these symptom categories, our patients experienced all of the general categories except for blood abnormalities and menstrual symptoms (our patients were males).

## Conclusion

This case series provides an opportunity to review the need for safe handling of old munitions, and associated findings in an exposure population. This topic remains important because of the very large quantities of old munitions still waiting to be demilitarized. Weaknesses in our report include the workers’ compensation setting. In particular, the fractious relationship between affected workers and worker health insurers limited follow-up during the period in which we would have expected symptoms and laboratory findings to completely resolve.

We do not know if personnel proposing the beneficial use in blasting had investigated the known toxicity of tetryl exposure or the associated needs for safe handling. While extensive, this literature is old and generally difficult to find. The concept of using former munitions for beneficial use in blasting is not necessarily bad. The problems experienced by these workers came from some combination of the absence of knowledge about the known toxicity of the material and absence of exposure controls. Modern explosives are designed to have a much lower health risk from workplace exposure, and most can be handled without dermal and respiratory protective gear. The historical knowledge of the needs of explosives workers and demilitarizing workers who handle older munitions can clearly be forgotten and neglected, with attendant health and operational consequences.

### Ethics approval and consent to participate

The IRB WVU Institutional Review Board (IRB) judged this protocol (“Case Series of Tetryl-exposed workers”) to be exempt. (Tracking # H-20286).
